# Supplementary material for: The interactome of intact mitochondria by cross-linking mass spectrometry provides evidence for coexisting respiratory supercomplexes
Source: Mol Cell Proteomics. 2017 Dec 8;17(2):216–32. doi: 10.1074/mcp.RA117.000470 (PMC5795388; doi:10.1074/mcp.RA117.000470)
Supplement: Supplemental Data [file supp_RA117.000470_133922_0_supp_23978_fzffwf.zip › spectra_annotation/mito_DR_spectra_annotation/356-1-1-1-2-1.pdf]

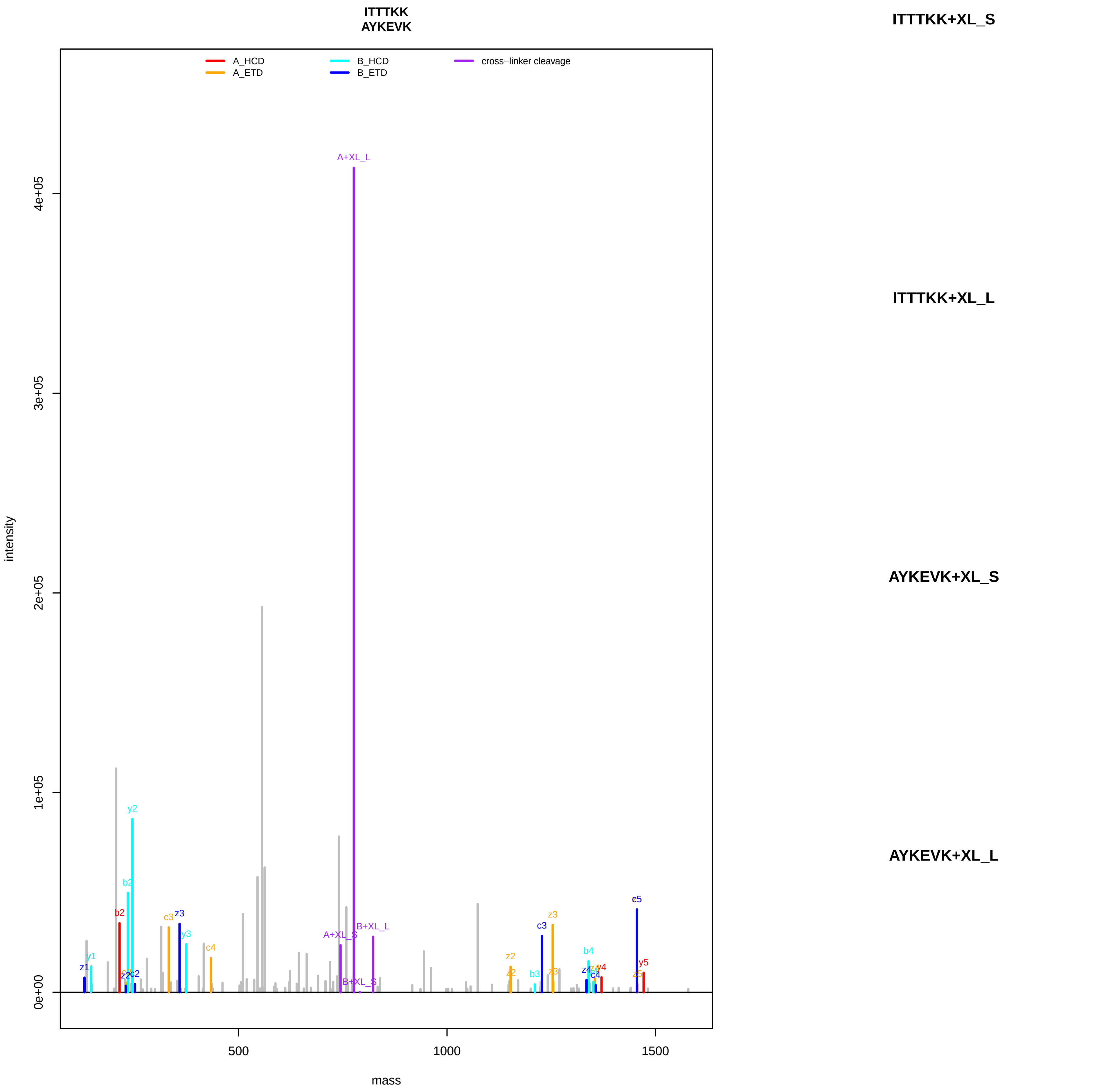
ITTTKK
AYKEVK
A_HCD
A_ETD
B_HCD
B_ETD
cross−linker cleavage
A+XL_L
A+XL_S
B+XL_L
B+XL_S
b2
y1
y2
y3
z1
z2
c2
c3
c4
z3
b3
b4
c5
y4
y5
z4
intensity
0e+00
1e+05
2e+05
3e+05
4e+05
mass
500
1000
1500
ITTTKK+XL_S
ITTTKK+XL_L
AYKEVK+XL_S
AYKEVK+XL_L
